# Enrichment of Root Endophytic Bacteria from Populus deltoides and Single-Cell-Genomics Analysis

**DOI:** 10.1128/AEM.01285-16

**Published:** 2016-08-30

**Authors:** Sagar M. Utturkar, W. Nathan Cude, Michael S. Robeson, Zamin K. Yang, Dawn M. Klingeman, Miriam L. Land, Steve L. Allman, Tse-Yuan S. Lu, Steven D. Brown, Christopher W. Schadt, Mircea Podar, Mitchel J. Doktycz, Dale A. Pelletier

**Affiliations:** a Biosciences Division, Oak Ridge National Laboratory, Oak Ridge, Tennessee, USA; b Graduate School of Genome Science and Technology, University of Tennessee, Knoxville, Tennessee, USA; University of Bayreuth

## Abstract

Bacterial endophytes that colonize Populus trees contribute to nutrient acquisition, prime immunity responses, and directly or indirectly increase both above- and below-ground biomasses. Endophytes are embedded within plant material, so physical separation and isolation are difficult tasks. Application of culture-independent methods, such as metagenome or bacterial transcriptome sequencing, has been limited due to the predominance of DNA from the plant biomass. Here, we describe a modified differential and density gradient centrifugation-based protocol for the separation of endophytic bacteria from Populus roots. This protocol achieved substantial reduction in contaminating plant DNA, allowed enrichment of endophytic bacteria away from the plant material, and enabled single-cell genomics analysis. Four single-cell genomes were selected for whole-genome amplification based on their rarity in the microbiome (potentially uncultured taxa) as well as their inferred abilities to form associations with plants. Bioinformatics analyses, including assembly, contamination removal, and completeness estimation, were performed to obtain single-amplified genomes (SAGs) of organisms from the phyla Armatimonadetes, Verrucomicrobia, and Planctomycetes, which were unrepresented in our previous cultivation efforts. Comparative genomic analysis revealed unique characteristics of each SAG that could facilitate future cultivation efforts for these bacteria.

**IMPORTANCE** Plant roots harbor a diverse collection of microbes that live within host tissues. To gain a comprehensive understanding of microbial adaptations to this endophytic lifestyle from strains that cannot be cultivated, it is necessary to separate bacterial cells from the predominance of plant tissue. This study provides a valuable approach for the separation and isolation of endophytic bacteria from plant root tissue. Isolated live bacteria provide material for microbiome sequencing, single-cell genomics, and analyses of genomes of uncultured bacteria to provide genomics information that will facilitate future cultivation attempts.

## INTRODUCTION

Microorganisms are the most phylogenetically diverse and abundant life forms on earth, yet an in depth understanding of their individual physiological diversities was largely limited to the species that can be grown in culture until the advent of cultivation independent methods (1, 2). The presence of many groups of yet uncultured bacteria was revealed mainly through cultivation-independent molecular surveys based on conserved marker genes (small subunit ribosome component, or 16S rRNA) (3). According to 16S rRNA-based phylogeny, microbial species fall into 60 major descents (phyla or divisions) within the bacterial and archaeal domains, of which half have no cultivated representatives (1). Conventional approaches to bring this uncultured majority of bacteria into pure culture are limited by the ability to mimic the required nutrients and microenvironment conditions. Modern cultivation approaches include the use of microfluidics chips (4), the recent iChip design to cultivate microbes in their natural environments (5), or inferred phenotypic traits for the selection of effective cultivation conditions (6, 7). Despite a few successes achieved through such intensive approaches, the large majority of microorganisms yet remain uncultured to such a large extent that this majority has often been referred to as microbial dark matter (8).

An alternative approach to study such intractable organisms is to bypass the culturing altogether and instead infer function from DNA by direct sequencing methods. Metagenomics, or direct sequencing of DNA from mixed environmental samples, can be applied to address the problem of such uncultured microbes (9); in some cases, draft or even complete genomes of the uncultured bacteria have been recovered, computationally segregated into individual taxa or populations, and assembled solely from metagenomics data (10–12). A complementary culture-independent approach for obtaining genomes from uncultured microbes is single-cell genomics (SCG). This approach involves amplification and sequencing of DNA from single cell or a few cells obtained directly from environmental samples separated by flow cytometry or other methods (13). The SCG approach could sometimes be advantageous over metagenomics sequencing for targeted recovery of genomes. In particular, natural populations that are present in low abundance or samples with high degrees of genomic heterogeneity may be more accessible through SCG than through metagenomics. The power of the SCG approach was demonstrated by a recent study in which 200 single cells were isolated from different habitats, including Nevada hot spring sediments and water from near hydrothermal vents in the Pacific Ocean. The researchers sequenced the genome of each cell and classified the cells into more than 20 new archaeal and bacterial lineages without any cultivated representatives (1). Many large-scale studies, including the Microbial Earth Project (generation of comprehensive genome catalogue of all archaeal and bacterial type strains) and the Human Microbiome Project (sequencing uncultured bacteria from the human microbiome), have relied at least in part on SCG approaches.

Efforts to understand the dynamic interface that exists between plants, the environment, and their microbiomes are critical for biofuel production, agricultural, and environmental sustainability. The soil surrounding the roots of plants accommodates an abundance of microorganisms due to the presence of nutrient-rich plant-derived exudates. The interface between plant root and soil constitutes the rhizosphere (14), and the inside of the root tissues constitutes the endosphere environment (15). These two compartments represent distinct environments for the growth of microbes. Both culture-independent and culture-dependent assessments of microbial communities from Populus have been undertaken, which includes community profiling using phylogenetic marker genes (16–18) and large culture collections of endosphere and rhizosphere isolates (19–21). The microbiome in these root-associated environments is comprised primarily of bacteria and fungi and, to a lesser extent, archaea which are virtually absent from the endosphere (18). Each of these may have potentially beneficial, neutral, or detrimental effects on plant growth and development. Microorganisms within the plant endosphere and rhizosphere are metabolically diverse (22–24) and can promote plant growth by fixing atmospheric nitrogen, solubilizing inorganic phosphorus, increasing the availability of nitrogen sources, producing plant phytohormones, decreasing ethylene stress, suppressing pathogens, and inducing systemic resistance (25–30). Within the rhizosphere, bacterial concentrations can be as high as 10^9^ cells/g of soil (27). A phylogenetically distinct portion of the soil and rhizosphere populations is able to cross into the root and comprise the bacterial endosphere (18). Endophytic bacterial populations can be as high as 10^8^ cells/g of root material (27), but most often they are several orders of magnitude less, at 10^4^ of 10^5^ cell/g of root. Because of the close association between endophytic bacterial communities and host tissues, physical separation of the microorganisms is a challenging task, and certain endophytic groups have been difficult to isolate and culture in a laboratory setting. Culture-independent methods have revealed the information about the uncultured endophytes and their phylogenetic diversities. However, application of metagenomics or SCG methods to interrogate endophytic samples has been difficult due to the prevalence of contaminating plant material and DNA. In this study, we describe a protocol for the enrichment of endophytic bacteria from Populus deltoides roots, upstream of cultivation and isolation, which in turn achieves reduction in host plant material and facilitates single-cell genomics analysis. In a first demonstration, we report on the genomes of organisms within the Armatimonadetes, Verrucomicrobia, and Planctomycetes that were absent in our previous cultivation efforts.

## MATERIALS AND METHODS

### Root harvesting.

Three Populus deltoides saplings were harvested from a field on the Oak Ridge National Laboratory campus (35°55′20.2″N, 84°19′24.4″W). Whole root samples were collected from each tree, and roots ≤5 mm in diameter were separated for enrichment. Total root weights used for enrichment were ∼10 g. The roots were cut into 1- to 2-cm-long pieces and placed into a 300-ml sterile flask with 40 ml of autoclaved Milli-Q water. The flasks were shaken at 200 rpm for 1 min, and the liquid was poured through a sterile miracloth (EMD Millipore, Billerica, MA) and collected in a 50-ml conical tube. Then, 100 ml of sterile Milli-Q water was added to the flasks containing the roots, and the flask was placed in a water bath sonicator at 40 kHz (Branson 2510; Danbury, CT) for 5 min to remove the rhizoplane microorganisms. The liquid was again poured through sterile miracloth and collected in a 50-ml conical tube. The two washes were pooled for each tree and represented the rhizosphere samples. The roots were further washed with sterile Mill-Q four more times, and the liquid was discarded. An ethanol and UV-sterilized (15 min) grinder (KSM2; Braun, Kronberg, Germany) was used to disrupt and homogenize the root samples in 40 ml of sterile Milli-Q. The homogenate was poured through sterile miracloth and collected in a 50-ml conical tube. This root homogenate constituted the endosphere sample.

### Differential and density centrifugation for microbial enrichment.

Microbes were enriched using an adaptation of a previously described method developed by Ikeda et al. (31, 32). Prior to the enrichment, 1 ml of the rhizosphere and of the endosphere samples was saved as an unenriched control for sequencing. The endosphere homogenates and the rhizosphere samples were centrifuged at 500 × *g* for 5 min at 10°C (Spinchron R; Beckman Coulter, Brea, CA). The supernatants were transferred to new conical tubes and centrifuged at 5,500 × *g* for 20 min at 10°C (Sorvall Evolution RC; Thermo Scientific, Carlsbad, CA). The supernatants were discarded, and the pellet was resuspended in 40 ml of bacterial cell extraction (BCE) buffer (50 mM Tris-HCl [pH 7.5] and 1% Triton X-100). The suspension was filtered through a layer of sterile miracloth and transferred to a sterile 50-ml Oak Ridge tube (Nalgene, Rochester, NY). The suspensions were centrifuged at 10,000 × *g* for 10 min at 10°C. The supernatants were discarded, and the pellet was resuspended in 40 ml of BCE buffer and filtered through a layer of sterile miracloth. The filtrate was centrifuged again at 10,000 × *g* for 10 min at 10°C. The supernatant was discarded, and the pellet was resuspended in 6 ml of 50 mM Tris-HCl (pH 7.5). The suspension was overlaid on 4 ml Histodenz (Sigma-Aldrich, St. Louis, MO) solution (8 g Histodenz dissolved in 10 ml of 50 mM Tris-HCl [pH 7.5]) in 10-ml ultra-clear centrifuge tubes (Beckman, Palo Alto, CA) such that the two solutions did not mix. The density centrifugation was run at 10,000 × *g* for 40 min at 10°C (Optima LE-80K; Beckman Coulter, Brea, CA). The microbial fraction (∼1 ml) was visible as a white band at the Histodenz-water interface. The microbial fraction was collected and washed by centrifugation at 10,000 × *g* for 3 min, followed by removal of the supernatant and resuspension of the pellet in 1 ml of 50 mM Tris-HCl (pH 7.5). Half of the sample was pelleted by centrifugation and stored at −20°C for DNA extraction. Glycerol at a final concentration of 25% (vol/vol) was added to the other half of the sample, and this sample was stored at −80°C for single-cell sorting.

### DNA extraction for microbiome sequencing.

DNA for the enriched and unenriched rhizosphere samples was extracted using the PowerSoil DNA isolation kit (Mo Bio Laboratories, Carlsbad, CA) using the provided protocol. DNA for the enriched and unenriched endosphere samples was extracted using the PowerPlant Pro DNA isolation kit with phenolic removal protocol (Mo Bio Laboratories, Carlsbad, CA) using the provided protocol.

### Sequencing, quality control, and analysis of paired-end Illumina data.

Libraries were prepared for the enriched endosphere DNA samples. Paired-end sequencing of the V4 region of the bacterial rRNA was performed on the Illumina MiSeq platform (San Diego, CA) using the protocol of Lundberg et al. (33). Sequence processing and quality control were performed through the use of the UPARSE, QIIME, and cutadapt pipelines (34, 35), as per Andrei et al. in 2015 (36), with the following modifications: reference-based chimera checking was performed with −minh 1.5. Low read count operational taxonomic units (OTUs) were removed using the command QIIME command filter_otus_from_otu_table.py –min_count_fraction 0.00005. Finally, enrichment of OTUs was determined via the use of the QIIME script group_significance.py and reported using false discovery rate (FDR)-adjusted *P* values.

### Single-cell sorting, multiple displacement amplification, and 16S rRNA Sanger sequencing.

The enriched microbial samples were stained with 5 μM Syto 9 nucleic acid stain (Life Technologies, Grand Island, NY). The stained samples were sorted on a Cytopeia Influx cell sorter (BD, Franklin Lakes, NJ) according to a previously published method (37). A flow cytometry plot was generated from forward scatter and green fluorescence. Ten gates were chosen from different positions on the plot. Single cells from enriched rhizosphere and endosphere samples from one tree were sorted into 20 96-well plates (10 plates from the rhizosphere and 10 plates from the endosphere; 1 plate each per gate).

The single-cell sorted plates were stored at −80°C prior to whole-genome amplification by multiple displacement amplification (MDA), as published previously (37). Briefly, cells were lysed by 3 μl of a buffer of 0.13 M KOH, 3.3 mM EDTA (pH 8.0), and 27.7 mM dithiothreitol (DTT) and heated to 95°C for 30 s. The reaction mixtures were immediately placed on ice for 10 min and then neutralized by the addition of a buffer of 0.13 M HCl, 0.42 M Tris (pH 7.0), and 0.18 M Tris (pH 8.0). The MDA was performed by adding 11 μl to each well of a reaction solution of 90.9 μM random hexamers with two protective phosphorothioate bonds on the 3′ end (Integrated DNA Technologies, Coralville, IA, USA), 1.09 mM deoxynucleoside triphosphates (dNTPs; Roche Indianapolis, IN, USA), 1.8× phi29 DNA polymerase buffer (New England BioLabs, Ipswich, MA, USA), 4 mM DTT (Roche), and ∼100 U phi29 DNA polymerase enzyme (purified in house). The MDA was performed in a thermocycler at 30°C for 10 h followed by inactivation at 80°C for 20 min. Plates were stored at −20°C.

For 16S rRNA sequencing of amplified DNA, 1 μl of the MDA was diluted into 150 μl of PCR-grade water. The remainder of the MDA was stored at −20°C. Universal 16S rRNA primers 27f (5′-AGAGTTTGATCMTGGCTCAG-3′) and 1492r (5′-TACGGYTACCTTGTTACGACTT-3′) were used to PCR amplify (in 50-μl reaction mixtures: 1× *Pfu* buffer, 200 μM dNTPs, 2 mM MgCl_2_, 5 μg bovine serum albumin, 300 μM forward and reverse primers, 0.2 μl *Pfu* polymerase, 37.90 μl double-distilled water (dH_2_O), and 1 μl 1:150 MDA product) the majority of the 16S rRNA sequences. Conditions for the PCR were 94°C for 2 min followed by 30 cycles of 94°C for 30 s, 55°C for 30 s, and 72°C for 2 min, with a final extension at 72°C for 5 min. Positive amplifications were identified by gel electrophoresis (1.5% agarose [wt/vol]). Positive PCR products were purified with PCR filtration plates (Millipore, Billerica, MA). The purified 16S rRNA products were sequenced by fluorescent dye-terminator cycle Sanger sequencing at the University of Tennessee Molecular Biology Resource Facility. Phylogenetic identifications were acquired using Ribosomal Database Project (RDP) classifier (38), SILVA incremental aligner (39), and NCBI blastn.

### Whole-genome amplification and sequencing of single cells.

Single-cell genomes were selected for whole-genome amplification based on 16S rRNA assignment. Nextera XT sequencing libraries (Illumina, La Jolla, CA) were prepared according to the manufacturer's recommendations (Part 15031942 Rev. E), stopping after library validation. In short, samples were fragmented, barcodes were appended, and samples were amplified. Libraries were cleaned using AMPure XP beads (Beckman Coulter, Indianapolis, IN). Final libraries were validated on an Agilent bioanalyzer (Agilent, Santa Clara, CA) using a DNA7500 chip, and concentration was determined on a Qubit with the broad-range double-stranded DNA assay (Life Technologies, Grand Island, NY). Libraries were prepared for sequencing following the manufacturer's recommended protocols. The library was denatured with 0.2 N sodium hydroxide and then diluted to the final sequencing concentration (19 pM). Libraries were loaded into the sequencing cassette (v3), and a paired-end (2-by-300) run was completed on an Illumina MiSeq instrument to obtain single amplified genomes (SAGs).

### Single-cell assembly.

Demultiplexed Illumina reads from the MiSeq software output were preprocessed using two separate approaches: (1) khmer digital normalization (40) and (2) regular assembly (41). The khmer digital normalization is a routinely applied method to SCG data in order to decrease the memory and time requirements for *de novo* assembly without significant impact on the assembly contents. The khmer protocol removes the redundant sequence reads, decreases sampling variation, removes the majority of errors, and substantially reduces the size of the sequence data (40). On the other hand, the regular assembly protocol utilized the complete set of raw reads without any data reduction. During regular assembly protocol, the quality trimming and filtering of raw sequence reads were performed for each SAG using CLC Genomics Workbench (CLC) (version 7.5.2) at a quality cutoff value of 0.02 (42). *De novo* genome assembly for each data set (khmer normalized and CLC trimmed) was performed using four assembly software packages with default options: IDBA-UD (version 1.1.1) (43), SPAdes (version 3.1.0) (44), Velvet-SC (version 0.7.62) (45), and CLC.

### Single-cell sequence contamination screening.

A number of recommended filtering operations (46) were performed to search for contaminated contigs. The first step was to check for any rRNA sequences from assembled SAGs, and blastn was performed to verify that they originated from a target organism of interest. A blastx search was performed against an NCBI-nonredundant database, and any contigs that matched (over half the contig length) with eukaryotic organisms were discarded. GC content was determined for each contig, and any that were outside a ±10% GC content range of the target organism were marked for removal. Cross-contamination between SAGs was analyzed by conservative searching of all assemblies against each other using blastn. Sequence regions that had more than 99.5% identity over at least 5,000 bp with another single cell were removed from the smaller contigs. Additionally, phylogenetic distribution of the genes on all removed contigs was manually reviewed to identify any false positives. The initial annotation of the screened single-cell genomes was performed using the annotation pipeline at Oak Ridge National Laboratory (47), and any contigs that did not contain protein-coding genes were discarded. The quality of the contamination-screened assemblies was verified using kmer frequency analysis (with settings: fragment window, 1,000 bp; fragment step, 200 bp; oligomer size, 4; minimum variation, 10) before and after contamination removal. Contamination-screened assemblies for each SAG were then submitted to the Integrated Microbial Genomes Expert Review (IMG-ER) system (48) for gene prediction and annotation.

### Genome completeness estimation.

The assembly completeness estimation was performed using the checkM tool (49) and the genome quality scoring matrix (50) with default parameters.

### Genome-based phylogenetic tree construction.

Universally distributed single-copy marker genes (51) were identified from individual SAGs. NCBI blastn was employed to extract these genes from other organisms within same phylogenetic lineage. For concatenated tree construction, all marker gene sequences extracted from the single organism were renamed per the organism name, e.g., all marker genes extracted from SAG E9H3 were named SAG E9H3. Individual marker genes from different organisms were collected into a single group, e.g., all marker genes corresponding to ribosomal protein L18 were collated as a single group (file) of fasta formatted sequences. Then, 18 files were created, corresponding to 18 commonly used conserved marker genes (see Table S1 in the supplemental material) from our SAGs and selected reference genomes from same phylum and imported into Geneious software (version 9.1.2). Multiple-sequence alignment for each individual group (file) was created using the MUSCLE alignment option, with a maximum of 8 iterations allowed. Individual alignments for 18 groups were sorted by high to low percentage pairwise identity and concatenated using the concatenate sequences or alignments tool from Geneious software. A maximum likelihood-based bootstrapped phylogenetic tree of concatenated sequence alignment was constructed using the PHYML tree builder plugin within Geneious software with the following options: substitution model, Blosum62; branch support, Bootstrap; number of bootstraps, 100; and optimized for topology/length/rate with topology search option Best (i.e., best of nearest-neighbor interchange [NNI] and subtree pruning and regrafting [SPR] search).

### Functional characterization of SAGs.

Genome statistics and comparative analyses were performed using various IMG-ER tools (52). The IMG annotation pipeline is integrated with a phenotype prediction tool (52) which generates phenotypes/metabolism assertions from pathways and was used to identify specific genome characteristics. The IMG pipeline also provided lists of protein-coding genes connected to transporter classification, KEGG pathways, and biosynthetic clusters that were used for functional characterization. The complete list of description/annotation for the Pfam clans (53) and the cluster of orthologous groups (COG) categories (54) is available at the IMG website. The abundance profile tool was employed to create functional profiles (containing COG categories and Pfam clans) for each of the SAGs and their corresponding draft/finished genomes. The abundance profile from the genomes contained a number of predicted genes for each COG/Pfam category, and clusters were identified that were uniquely present in SAGs but not close relatives. Another IMG tool, pathway via KEGG orthology (KO) terms, was used to identify the presence/absence of specific genes within pathways.

### Accession number(s).

Assembled and annotated SAGs are available on the IMG website with identification numbers 2626541630 (SAG R9F7), 2626541631 (SAG E9H3), 2626541627 (SAG E1D9), and 2626541629 (SAG E2G8). Raw data for 16S sequencing is available through the NCBI Sequence Read Archive (accession number SRP077616).

## RESULTS AND DISCUSSION

### Enrichment and analysis of endophytic bacteria.

**FIG 1:**
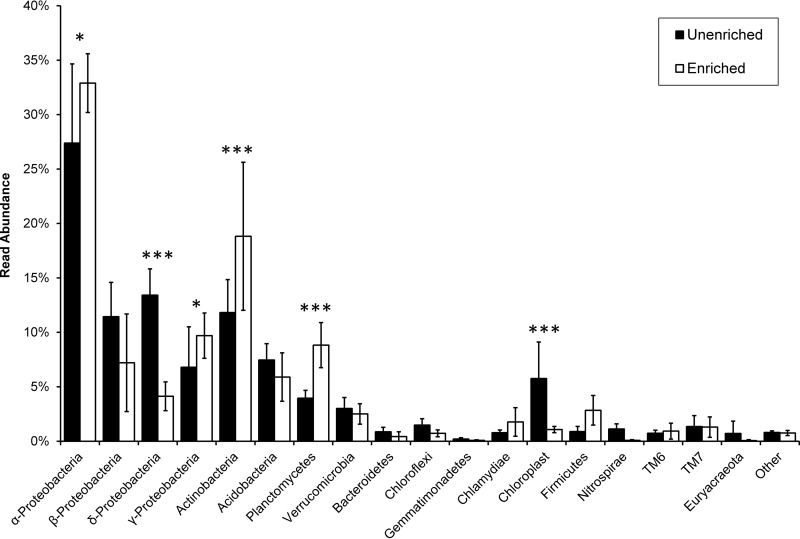
Comparison of bacterial 16S rRNA read abundance percentage, at the phylum level, between enriched and unenriched endosphere samples. Enrichment significance was determined via the use of the QIIME script group_significance.py and reported using FDR-adjusted *P* values. *** and *, *P* < 0.01 and *P* < 0.1, respectively.

Approximately 10^7^ to 10^8^ cells were enriched from the rhizosphere and endosphere samples using the current method (data not shown). On average, 33.67 ± 7.07 ng of DNA was isolated from the enrichments. In contrast, unenriched endosphere extractions yielded an average of 605.25 ± 469.84 ng of DNA, most of which was presumably from the host plant. The 16S rRNA phylotyping performed on the three enriched and three unenriched endosphere samples demonstrated that Proteobacteria dominated the endosphere of these saplings. These data showed similar read percent abundances at the phylum level, though some significant differences existed (Fig. 1). Phyla that were significantly increased in read abundance percentage in the average enrichment of the three trees were the Actinobacteria and the Planctomycetia (*P* < 0.01, FDR corrected). The Proteobacteria showed different enrichment profiles at the class level. Alphaproteobacteria and Gammaproteobacteria were significantly increased in read abundance percentage (*P* < 0.1, FDR corrected). Betaproteobacteria showed no significant difference, while Deltaproteobacteria were significantly decreased in read abundance percentage (*P* < 0.01, FDR corrected). Differences in read abundances between enriched and nonenriched samples could be due to several issues. Not all bacteria are captured by the enrichment. Bacteria that are tightly associated with plant material could be lost, as they would be removed with the plant fraction in filtering and centrifugation. Lysis during the enrichment could also change the sequencing read abundance, both positively, with more free DNA to sequence, and negatively, if that free DNA became degraded prior to sequencing. Importantly, contaminating chloroplast reads from the roots were significantly decreased in the enrichment by approximately 10-fold (∼7% to ≤0.7% of all reads; *P* < 0.01, FDR corrected) due to removal of plant material.

### Single-cell sorting, MDA amplification, and sequencing.

For single-cell sorting, the endosphere and rhizosphere enrichments from one tree were chosen, and cells from each sample were sorted into 10 96-well plates from 10 different gates on the cytometry plot. After MDA whole-genome amplification and 16S rRNA gene PCR amplification, there were 169 positive 16S rRNA gene amplifications (86 from the endosphere and 83 from the rhizosphere) based on agarose gel observations. PCR investigations of wells that did not produce bacterial 16S rRNA gene signals suggested that a further 179 wells may have contained fungal cells (data not shown). Of the 169 positive 16S rRNA signals, 115 were successfully sequenced by the Sanger method. The RDP classifier (38) and the NCBI reference RNA database were used to assign phylogeny to the amplified signals. Sorted cells represented multiple phyla, including Acidobacteria, Actinobacteria, Armatimonadetes (formally OP10), Bacteroidetes, Firmicutes, Planctomycetes, Proteobacteria, and Verrucomicrobia. Several 16S rRNA sequences appeared to represent members of the human microbiome, with sequences corresponding to Corynebacterium spp., Propionibacterium acnes, and Staphylococcus epidermidis, implying some potential skin contamination. It is unclear where this contamination originated, as care was taken to avoid contamination during the harvest and preparation of the samples; however, these are common contaminants in many studies (55). OTUs of these sequences were present in the 16S rRNA gene phylotyping data, though at low abundances (data not shown). Regardless, novel 16S rRNA sequences (<97% identity to sequenced relatives) from multiple phyla were present in the sorted cells. Four single-cell genomes were selected for whole-genome sequencing based on representing rare and uncultured phyla (from the NCBI database), abundance of OTUs present within Populus rhizosphere, and their inferred ability to form associations with plant. The 16S rRNA gene sequences from these single cells analyzed by the blast search algorithm revealed greater than 99% identity to Zavarzinella sp. (2 SAGs), Armatimonadetes sp., Acidobacteria sp., and Verrucomicrobia sp. that had previously been observed in microbiome studies of Populus endospheres (16–18) but that were not present in our culture collections from these systems.

### Genome assembly.

*De novo* genome assembly of single cells was performed using two data preprocessing approaches (khmer digital normalization and regular assembly) and four assembly software packages (SPAdes, Velvet-SC, IDBA-UD and CLC), as described in Materials and Methods. Independent of preprocessing approach, the IDBA-UD assembler always generated the best assembly statistics with the highest *N*_50_ values and total genome size assembled. It is worth mentioning that, although khmer normalization has become a prevalent step during single cell assembly, the khmer authors have prepared a blog about the application of the khmer protocol (http://ivory.idyll.org/blog/why-you-shouldnt-use-diginorm.html) which clearly suggests that normalization steps are not necessary when comparable results are obtained through regular assembly protocols. Our single-cell assemblies have comparable statistics from both khmer and regular assembly protocols. Based on the recommendations from the blog, IDBA-UD assemblies generated with regular assembly protocols were used for further downstream analysis.

### Contamination screening of single-cell amplified genomes.

Single-cell sequence data are often found to be contaminated with organisms other than the target population, and contamination removal is a necessary step (56). Contamination screening was performed as described in Materials and Methods. Four SAGs (identification numbers E1D9, E2G8, E9H3, and R9F7) contained 30 to 40% contaminants and generated assembly sizes of 4 to 7 Mb. Most of the contaminating DNA corresponded to eukaryotic lineages, with high similarity to human and plant species. The kmer frequency distribution graphs were created before and after contamination removal steps. Before contamination removal, there were two distinct kmer frequency clouds observed, and one of them (belonging mostly to DNA of eukaryotic origin) was absent after contamination removal, suggesting that we were able to effectively remove the majority of contaminants. Detailed assembly statistics for each SAG after contamination removal are presented in Table 1.

**TABLE 1:** Genome assembly statistics for each assembled SAG

SAG identification	Associated lineage	Postcontamination removal assembly statistics				CheckM statistics	
		No. of contigs	Maximum contig size (kb)	*N*_50_ contig size (bp)	Genome size (Mb)	Estimated completeness (%)	Estimated contamination (%)
E2G8	Armatimonadetes	366	61	10,515	2.3	27.80	0.19
E1D9	Verrucomicrobia	667	61	11,690	2.9	25.33	1.96
E9H3	Planctomycetes	837	110	17,338	5.7	51.32	1.51
R9F7	Planctomycetes	1,344	79	8,671	6.5	48.72	0.10

### Genome-based phylogenetic inference.

**FIG 2:**
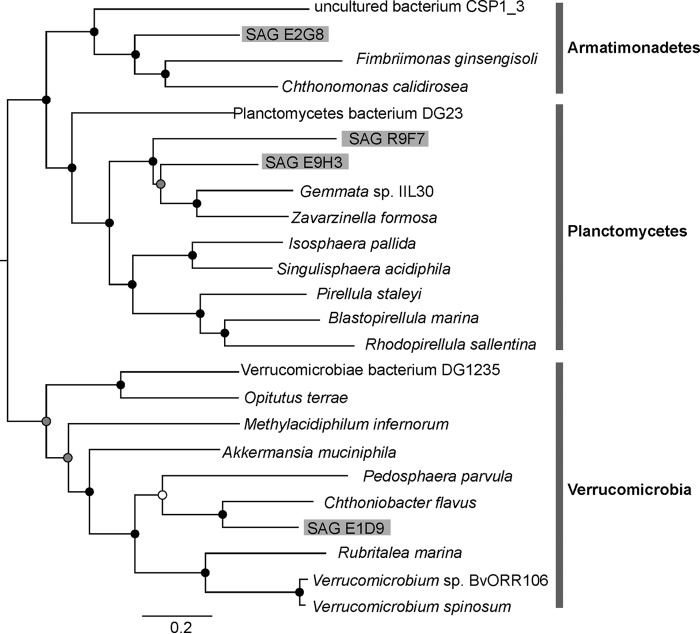
Phylogenetic analysis of generated SAGs. A bootstrapped maximum likelihood tree created by concatenation alignment of 18 commonly used marker genes is shown. Bootstrap values are indicated by colored dots on each node: black dots (80 to 100), gray dots (50 to 80), and white dots (<50).

Small-subunit (SSU) rRNA trees are well known predictors of phylogenetic novelty (57, 58). However, concatenated alignment of multiple universally distributed single-copy marker genes provides greater phylogenetic resolution than any individual gene for estimating a species tree (59). We constructed a bootstrapped maximum likelihood tree based on concatenation of 18 commonly used conserved marker genes that were present in most of our SAGs and selected reference genomes from the same phylum present on IMG (see Table S1 in the supplemental material). Phylogenetic analyses of the 18 gene concatenated alignments (Fig. 2) showed the presence of 3 distinct clusters corresponding to the phyla Armatimonadetes, Planctomycetes, and Verrucomicrobia, each supported by high bootstrapped values (>90). Each SAG in the analyses grouped with the members from their predicted lineages. The closest relatives for SAGs E9H3 and R9F7 were Zavarzinella formosa strain A10, a type strain of Zavarzinella formosa, and Gemmata sp. IIL30, respectively, from the Planctomycetes phylum. The closest relative for SAG E1D9 was Chthoniobacter flavus Ellin428 from the Verrucomicrobia phylum and for SAG E2G8 was Fimbriimonas ginsengisoli from the Armatimonadetes (formerly OP10) phylum. For additional verification, 16S rRNA sequences derived from assembly of each SAG were analyzed by sequence match and classifier tools (38) available at the RDP database (60) and found to be matching with expected lineages, thus confirming the origin for each SAG.

### Genome completeness analysis.

The checkM tool classified each SAG belonging to the domain Bacteria, with an estimated completeness of 27% for Armatimonadetes sp. SAG E2G8, of 25% for Verrucomicrobia sp. SAG E1D9, of 48% for Planctomycetes sp. SAG R9F7, and of 51% for Planctomycetes sp. SAG E9H3. Despite contamination removal steps, each SAG was determined to contain contaminants at very low levels (<2%). Detailed quality statistics determined by checkM tool are described in Table 1. Additional evaluation of the quality and completeness of the SAGs was performed by assessment of a set of essential genes present in each genome (50). By this method, the estimated completeness was 64% for Armatimonadetes sp. SAG E2G8, 54% for Verrucomicrobia sp. SAG E1D9, 68% for Planctomycetes sp. SAG R9F7, and 64% for Planctomycetes sp. SAG E9H3 (see Table S2 in the supplemental material). A combined quality score was assigned to each SAG based on the presence of essential gene sets and the completeness of rRNA and tRNA. The combined quality score was >0.6 for Armatimonadetes sp. SAG E2G8 and Planctomycetes sp. SAGs R9F7 and 0.36 for Verrucomicrobia sp. SAG E1D9 (see Table S2 in the supplemental material). The maximum score assigned by this matrix was 1, in which the complete set of all the essential genes, tRNA, and rRNA were present. These two tools provided independent evaluations for SAG quality estimations using different algorithms. The checkM tool used stringent parameters (ubiquitous and single-copy genes within a phylogenetic lineage, various genomic characteristics, and proximity within a reference genome tree) and provided robust estimations. These completeness estimation results were in accordance with a recent study which estimated genome completeness of 201 SAGs from uncultured archaeal and bacterial cells in the range of less than 10% to greater than 90%, with a mean of 40% (1). Another important factor is that these rare and uncultured small bacterial cells are known to be missing many so-called essential genes and core biosynthetic pathways and so are at least partially dependent on other community members (11, 61, 62). Therefore, the completeness estimation based on common ubiquitous genes from cultured bacteria may only be a relative measure. In another recent example, a near-complete genome of a Verrucomicrobia phylotype was reconstructed from metagenomic data which showed a drastic reduction (2.81 Mb compared to the predicted effective mean genome size of 4.74 Mb for soil bacteria) (63). Therefore, genome reduction could also be a possible reason for comparatively lower completeness estimation scores.

### Functional characterization of single cells.

The availability of genomic information for uncultured microbes that remain elusive to direct investigation enables comparative genomic analyses and allows inferences about biochemical properties and metabolic traits. These inferences are useful to predict the roles of these microbes in specific environments and could be used to select effective cultivation conditions. Comparisons between SAGs and corresponding finished/draft genomes revealed the presence of several unique genes and functional characteristics of individual SAGs, which allowed for the prediction of putative roles for these bacteria in the plant environment. The putative functional characteristics for individual SAGs compared to close relatives are described below.

### (i) SAG of the phylum Armatimonadetes.

**FIG 3:**
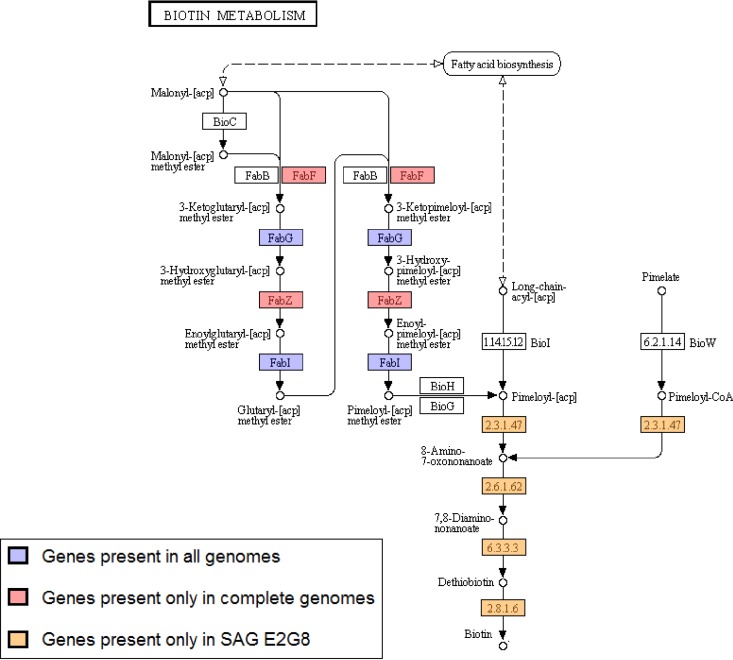
Summary of presence/absence of candidate genes for biotin biosynthesis pathway in Armatimonadetes sp. SAG E2G8 and complete genomes of Fimbriimonas ginsengisoli Gsoil 348 and Chthonomonas calidirosea T49, DSM 23976.

The Armatimonadetes sp. SAG E2G8 was isolated from the Populus endosphere, and its genome was compared with the complete genomes of the only two cultured members from the same phylum, Fimbriimonas ginsengisoli Gsoil 348 (IMG ID 2585427636) (64) and Chthonomonas calidirosea T49, DSM 23976 (IMG ID 2524614646) (65). One potentially key observation was the unique presence of biotin (vitamin B7) biosynthesis-related genes in SAG E2G8 compared to the two cultured representatives. Biotin biosynthesis starts with the metabolite malonyl-acyl carrier protein (ACP), which is converted to the precursor pimeloyl-ACP through a series of enzymatic reactions. Some bacteria also have an alternative route, in which the precursor pimeloyl-CoA is derived from pimelate (66). Pimeloyl-ACP and pimeloyl-coenzyme A act as precursor molecules, and conversion to biotin takes place through four reaction steps. Interestingly, the genes involved in the final four steps (8-amino-7-oxononanoate synthase [EC 2.3.1.47], 8-amino-7-oxononanoate aminotransferase [EC 2.6.1.62], dethiobiotin synthase [EC 6.3.3.3], and biotin synthase [EC 2.8.1.6]) were present only in our Armatimonadetes SAG and missing from the finished genomes. The final four steps in biotin biosynthesis pathway are known to be conserved among biotin-producing organisms (67), suggesting a possible biotin producing phenotype for Armatimonadetes sp. SAG E2G8. However, some intermediate genes involved in conversion of the starting metabolites (malonyl-ACP or pimelate) to precursor molecules were missing from the Armatimonadetes sp. SAG E2G8 (Fig. 3), possibly because the genome was incomplete or because the precursors could be obtained from within the plant endosphere.

The Armatimonadetes sp. SAG E2G8 contains 21 σ-70-like proteins and has a high σ factor-to-genome size (σ/Mb) ratio, as also reported for the Chthonomonas calidirosea strain T49 (65). The high-abundance σ factors are predicted to coordinate transcriptional regulation of functionally related but dispersed genes (65) and are likely to be involved in the transcription regulatory mechanism in SAG E2G8. Central metabolism appears to proceed via standard glycolysis and the tricarboxylic acid cycle, although some key genes were missing. The presence of genes related to oxidative phosphorylation supports a likely aerobic respiration phenotype. The SAG also contains genes for extracellular nitrate/nitrite transporters, assimilatory nitrate reductase (*narB*), and dissimilatory nitrate reduction components (*nirB*, *nirD*) involved in nitrogen cycling which could be beneficial inside and outside the plant. We also identified the genes coding for cyanate lyase (Ga0078968_13342) and carbonic anhydrase (Ga0078968_11235, Ga0078968_12064) in SAG E2G8, which might confer the ability to tolerate environmental cyanate.

### (ii) SAGs of the phylum Planctomycetes.

**FIG 4:**
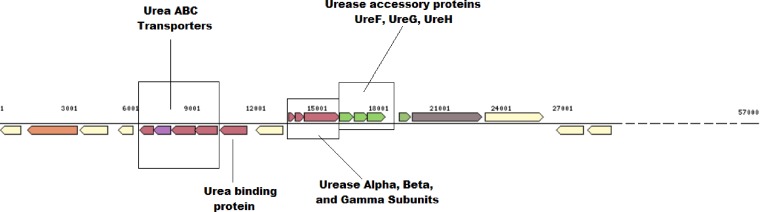
Diagram of the urease gene clusters present in the Planctomycetes sp. SAG E9H3. Various components of the gene cluster are indicated in separate boxes.

Two SAGs of the phylum Planctomycetes of endosphere (E9H3) and rhizosphere (R9F7) origins were compared with the draft genome of Zavarzinella formosa strain A10^T^ (IMG identification number 2548877000) (68), the closest sequenced relative based on 16S rRNA gene sequence similarity. The key distinction between the Planctomycetes SAGs and Zavarzinella formosa strain A10^T^ was the presence of the urease system as a unique feature of SAG E9H3. The urease gene cluster (including urease α, β, and γ subunits (Ga0078970_101213, Ga0078970_101212, and Ga0078970_101211) and urease accessory proteins UreF (Ga0078970_101214), UreG (Ga0078970_101215), and UreH (Ga0078970_101216) were detected as part of the operon on contig Ga0078970_1012 in SAG E9H3. Other accessory genes coding for the urea binding protein (Ga0078970_10129) and the urea ABC transporters (Ga0078970_10125, Ga0078970_10126) were also detected on the same contig and as part of the operon (Fig. 4). Active ureases require a nickel-containing active site to catalyze the hydrolysis of urea to ammonia and carbamate (69). We also identified the genes related to COG0378 with the predicted function of Ni2^+^-binding GTPase involved in regulation of expression and maturation of urease and hydrogenase in SAG E9H3, and these genes were missing from strain A10^T^. SAG E9H3 also contained the gene related to hydrogenase/urease accessory protein HupE (Ga0078970_115010), which is implicated as a secondary transporter for nickel or cobalt (70). Additionally, genes involved in various acid tolerance or pH homeostasis mechanisms, such as the F_1_F_0_-ATPase proton pump (71), the arginine and/or glutamate decarboxylase system (72, 73), and the urease system (74, 75), were present in SAG E9H3 and/or SAG R9F7, suggesting the presence of possible pH tolerance and regulation mechanism.

Most of the genes involved in glycolysis, the citric acid cycle, the pentose phosphate pathway, and pyruvate metabolism were identified in both SAGs and Zavarzinella formosa strain A10^T^, which suggests a common route for central metabolism. The IMG phenotype prediction tool (52) predicted an aerobic phenotype for the SAG E9H3 based on presence of the genes coding for cytochrome *bd*-I ubiquinol oxidase (Ga0078970_104513, Ga0078970_104514) which are known to be involved in ubiquinol oxidation. Interestingly, in the cytochrome *bd* complex, genes were detected only in E9H3 but were missing from strain A10^T^ and R9F7, though they could have been missing from R9F7 because the genome was incomplete. Pilus assembly-related genes were also present in both SAGs and might serve the function of cell-to-cell or surface attachment, as observed in case of Z. formosa strain A10^T^ (76). Further, a gene coding for putative pectate lyase was found in the rhizosphere SAG R9F7 that is indicative of a plant degradation lifestyle. Pectins are a major component of plant cell walls and an abundant carbon source in the rhizosphere (77).

### (iii) SAG of the phylum Verrucomicrobia.

The Verrucomicrobia sp. SAG E1D9 genome came from the Populus endosphere, and its SAG was compared against the draft genome sequence of its relative Chthoniobacter flavus Ellin428 (78). Most of the genes involved in glycolysis pathway, several genes involved in citric acid cycle, and those of the pentose phosphate pathway were present, suggesting a traditional route for carbon metabolism. Although a majority of the members of the phylum Verrucomicrobia exhibit aerobic phenotypes, many genes involved in oxidative phosphorylation were missing from the SAG E1D9, possibly because of the incomplete nature of the genome. A putative catalase gene (Ga0078966_11592) was present in both SAG E1D9 and Ellin428, though biochemical tests of Ellin428 revealed catalase negative activity (79). Based on the Pfam functional profile, a total of 39 protein-coding genes related to various glycosyl hydrolase families were identified, which included 6 genes corresponding to cellulases (glycosyl hydrolase family 5) and 12 genes corresponding to glycosyl hydrolase family 16. Members of this family are known to hydrolyze a variety of plant glucans and galactans. Twelve of these glycosyl hydrolase genes were found in the Verrucomicrobia sp. SAG E1D9 but not in Ellin428. The presence of various glycosyl hydrolase family-related genes in SAG E1D9 suggests the ability to degrade complex plant material and could indicate how the organism gained access to the endosphere.

### Strategies for bringing culture to the uncultured.

Culture-independent approaches have revolutionized our understanding of microbial diversity and evolution (10); however, laboratory cultures are essential for detailed investigations of complex organismal biology and core biosynthetic capacities and to infer specialized functions within communities. There have been examples of genome-informed isolation of novel microbes, in which sequence-derived information was useful to select appropriate cultivation conditions (6, 7, 80). Similarly, genomic information and characteristics described for current SAGs may be useful to select appropriate cultivation conditions. All of the SAGs described above share an isolation origin, the Populus root environment, which is rich in complex plant polysaccharides like cellulose, hemicellulose, and other complex heteropolysaccharides. Uncultured bacteria, predominantly diverse Planctomycetes, have been shown to be adapted to use these complex heteropolysaccharides for growth, followed by populations of Armatimonadetes and Verrucomicrobia as secondary consumers (81). The current SAGs of Planctomycetes and Verrucomicrobia contain a variety of glycoside hydrolase, polysaccharide, and pectate lyase genes, suggesting the possibility of a mechanism to scavenge a wide variety of plant oligosaccharides and polysaccharides. Therefore, the use of these complex heteropolysaccharides in a growth medium may provide a means for culturing these bacteria by reducing resource competition. The presence of the urease gene cluster and the additional pH tolerance mechanisms of Planctomycetes SAGs hint that growth media with extreme pH conditions and urea as a sole nitrogen source might further reduce nutrient competition. Similarly, the putative biotin biosynthesis ability of the Armatimonadetes SAG would suggest that growth media lacking biotin could limit the growth of biotin heterotrophs. Several of these conditions, including use of diluted, low-nutrient, low-pH media and use of a complex heteropolysaccharide as an energy source, were key to the successful cultivation of first member of phylum Armatimonadetes (OP10) (82) and may also facilitate future cultivation efforts for the organisms represented by these SAGs.

### Conclusion.

Physical separation and isolation of plant-associated bacteria from plant material are challenging tasks. Our modified enrichment protocol based on differential and density gradient centrifugation was able to achieve a significant reduction in contaminating plant debris and DNA and enriched for bacteria from the rhizosphere and endosphere. This protocol also enabled single-cell genomic analyses of enriched bacterial samples that generated genomes of previously uncultured bacteria of interest. Bioinformatics and comparative genomic analyses revealed the unique characteristics of these SAGs compared to their close relatives. The unique characteristics include the presence of the biotin biosynthesis gene cluster in Armatimonadetes SAG, the urease gene cluster in Planctomycetes SAGs, and the putative ability to degrade complex plant material in Verrucomicrobia SAG. This genomic information may facilitate future efforts to culture these bacteria. This study provides a modified enrichment protocol for the separation and isolation of a live endophytic bacterial sample and facilitates further analyses by single-cell genomics, metagenomics, or culture-based methods.

## Supplementary Material

Supplemental material

## References

[1] Rinke C, Schwientek P, Sczyrba A, Ivanova NN, Anderson IJ, Cheng JF, Darling A, Malfatti S, Swan BK, Gies EA, Dodsworth JA, Hedlund BP, Tsiamis G, Sievert SM, Liu WT, Eisen JA, Hallam SJ, Kyrpides NC, Stepanauskas R, Rubin EM, Hugenholtz P, Woyke T 2013 Insights into the phylogeny and coding potential of microbial dark matter. Nature 499:431–437. doi:10.1038/nature12352. 23851394

[2] Solden L, Lloyd K, Wrighton K 2016 The bright side of microbial dark matter: lessons learned from the uncultivated majority. Curr Opin Microbiol 31:217–226. doi:10.1016/j.mib.2016.04.020. 27196505

[3] Rajendhran J, Gunasekaran P 2011 Microbial phylogeny and diversity: small subunit ribosomal RNA sequence analysis and beyond. Microbiol Res 166:99–110. doi:10.1016/j.micres.2010.02.003. 20223646

[4] Seshadri R, Paulsen IT, Eisen JA, Read TD, Nelson KE, Nelson WC, Ward NL, Tettelin H, Davidsen TM, Beanan MJ, Deboy RT, Daugherty SC, Brinkac LM, Madupu R, Dodson RJ, Khouri HM, Lee KH, Carty HA, Scanlan D, Heinzen RA, Thompson HA, Samuel JE, Fraser CM, Heidelberg JF 2003 Complete genome sequence of the Q-fever pathogen Coxiella burnetii. Proc Natl Acad Sci U S A 100:5455–5460. doi:10.1073/pnas.0931379100. 12704232 PMC154366

[5] Ling LL, Schneider T, Peoples AJ, Spoering AL, Engels I, Conlon BP, Mueller A, Schaberle TF, Hughes DE, Epstein S, Jones M, Lazarides L, Steadman VA, Cohen DR, Felix CR, Fetterman KA, Millett WP, Nitti AG, Zullo AM, Chen C, Lewis K 2015 A new antibiotic kills pathogens without detectable resistance. Nature 517:455–459. doi:10.1038/nature14098. 25561178 PMC7414797

[6] Bomar L, Maltz M, Colston S, Graf J 2011 Directed culturing of microorganisms using metatranscriptomics. mBio 2:e00012-11. doi:10.1128/mBio.00012-11. 21467263 PMC3069634

[7] Tyson GW, Lo I, Baker BJ, Allen EE, Hugenholtz P, Banfield JF 2005 Genome-directed isolation of the key nitrogen fixer Leptospirillum ferrodiazotrophum sp. nov. from an acidophilic microbial community. Appl Environ Microbiol 71:6319–6324. doi:10.1128/AEM.71.10.6319-6324.2005. 16204553 PMC1266007

[8] Marcy Y, Ouverney C, Bik EM, Losekann T, Ivanova N, Martin HG, Szeto E, Platt D, Hugenholtz P, Relman DA, Quake SR 2007 Dissecting biological dark matter with single-cell genetic analysis of rare and uncultivated TM7 microbes from the human mouth. Proc Natl Acad Sci U S A 104:11889–11894. doi:10.1073/pnas.0704662104. 17620602 PMC1924555

[9] Handelsman J 2004 Metagenomics: application of genomics to uncultured microorganisms. Microbiol Mol Biol Rev 68:669–685. doi:10.1128/MMBR.68.4.669-685.2004. 15590779 PMC539003

[10] Hug LA, Baker BJ, Anantharaman K, Brown CT, Probst AJ, Castelle CJ, Butterfield CN, Hernsdorf AW, Amano Y, Ise K, Suzuki Y, Dudek N, Relman DA, Finstad KM, Amundson R, Thomas BC, Banfield JF 2016 A new view of the tree of life. Nat Microbiol 1:16048. doi:10.1038/nmicrobiol.2016.48. 27572647

[11] Luef B, Frischkorn KR, Wrighton KC, Holman HY, Birarda G, Thomas BC, Singh A, Williams KH, Siegerist CE, Tringe SG, Downing KH, Comolli LR, Banfield JF 2015 Diverse uncultivated ultra-small bacterial cells in groundwater. Nat Commun 6:6372. doi:10.1038/ncomms7372. 25721682

[12] Sharon I, Banfield JF 2013 Genomes from metagenomics. Science 342:1057–1058. doi:10.1126/science.1247023. 24288324

[13] Stepanauskas R 2012 Single cell genomics: an individual look at microbes. Curr Opin Microbiol 15:613–620. doi:10.1016/j.mib.2012.09.001. 23026140

[14] Rout ME, Callaway RM 2012 Interactions between exotic invasive plants and soil microbes in the rhizosphere suggest that everything is not everywhere. Ann Bot 110:213–222. doi:10.1093/aob/mcs061. 22451600 PMC3394644

[15] Turner TR, James EK, Poole PS 2013 The plant microbiome. Genome Biol 14:209. doi:10.1186/gb-2013-14-6-209. 23805896 PMC3706808

[16] Bonito G, Reynolds H, Robeson MS Jr, Nelson J, Hodkinson BP, Tuskan G, Schadt CW, Vilgalys R 2014 Plant host and soil origin influence fungal and bacterial assemblages in the roots of woody plants. Mol Ecol 23:3356–3370. doi:10.1111/mec.12821. 24894495

[17] Gottel NR, Castro HF, Kerley M, Yang Z, Pelletier DA, Podar M, Karpinets T, Uberbacher E, Tuskan GA, Vilgalys R, Doktycz MJ, Schadt CW 2011 Distinct microbial communities within the endosphere and rhizosphere of Populus deltoides roots across contrasting soil types. Appl Environ Microbiol 77:5934–5944. doi:10.1128/AEM.05255-11. 21764952 PMC3165402

[18] Shakya M, Gottel N, Castro H, Yang ZK, Gunter L, Labbe J, Muchero W, Bonito G, Vilgalys R, Tuskan G, Podar M, Schadt CW 2013 A multifactor analysis of fungal and bacterial community structure in the root microbiome of mature Populus deltoides trees. PLoS One 8:e76382. doi:10.1371/journal.pone.0076382. 24146861 PMC3797799

[19] Brown SD, Klingeman DM, Lu TY, Johnson CM, Utturkar SM, Land ML, Schadt CW, Doktycz MJ, Pelletier DA 2012 Draft genome sequence of Rhizobium sp. strain PDO1-076, a bacterium isolated from Populus deltoides. J Bacteriol 194:2383–2384. doi:10.1128/JB.00198-12. 22493196 PMC3347069

[20] Brown SD, Utturkar SM, Klingeman DM, Johnson CM, Martin SL, Land ML, Lu TY, Schadt CW, Doktycz MJ, Pelletier DA 2012 Twenty-one genome sequences from Pseudomonas species and 19 genome sequences from diverse bacteria isolated from the rhizosphere and endosphere of Populus deltoides. J Bacteriol 194:5991–5993. doi:10.1128/JB.01243-12. 23045501 PMC3486089

[21] Klingeman DM, Utturkar S, Lu TY, Schadt CW, Pelletier DA, Brown SD 2015 Draft genome sequences of four Streptomyces isolates from the Populus trichocarpa root endosphere and rhizosphere. Genome Announc 3:e01344-15. doi:10.1128/genomeA01344-15. 26564053 PMC4972787

[22] Bible AN, Fletcher SJ, Pelletier DA, Schadt CW, Jawdy SS, Weston DJ, Engle NL, Tschaplinski TJ, Masyuko R, Polisetti S, Bohn PW, Coutinho TA, Doktycz MJ, Morrell-Falvey JL 2016 A carotenoid-deficient mutant in Pantoea sp. YR343, a bacteria isolated from the rhizosphere of Populus deltoides, is defective in root colonization. Front Microbiol 7:491. 27148182 10.3389/fmicb.2016.00491 PMC4834302

[23] Jun SR, Wassenaar TM, Nookaew I, Hauser L, Wanchai V, Land M, Timm CM, Lu TY, Schadt CW, Doktycz MJ, Pelletier DA, Ussery DW 2016 Diversity of Pseudomonas genomes, including Populus-associated isolates, as revealed by comparative genome analysis. Appl Environ Microbiol 82:375–383. doi:10.1128/AEM.02612-15. 26519390 PMC4702629

[24] Timm CM, Campbell AG, Utturkar SM, Jun SR, Parales RE, Tan WA, Robeson MS, Lu TY, Jawdy S, Brown SD, Ussery DW, Schadt CW, Tuskan GA, Doktycz MJ, Weston DJ, Pelletier DA 2015 Metabolic functions of Pseudomonas fluorescens strains from Populus deltoides depend on rhizosphere or endosphere isolation compartment. Front Microbiol 6:1118. doi:10.3389/fmicb.2015.01118. 26528266 PMC4604316

[25] Abramovitch RB, Anderson JC, Martin GB 2006 Bacterial elicitation and evasion of plant innate immunity. Nat Rev Mol Cell Biol 7:601–611. doi:10.1038/nrm1984. 16936700 PMC2842591

[26] Berendsen RL, Pieterse CM, Bakker PA 2012 The rhizosphere microbiome and plant health. Trends Plant Sci 17:478–486. doi:10.1016/j.tplants.2012.04.001. 22564542

[27] Bulgarelli D, Schlaeppi K, Spaepen S, Ver Loren van Themaat E, Schulze-Lefert P 2013 Structure and functions of the bacterial microbiota of plants. Annu Rev Plant Biol 64:807–838. doi:10.1146/annurev-arplant-050312-120106. 23373698

[28] Lugtenberg B, Kamilova F 2009 Plant-growth-promoting rhizobacteria. Annu Rev Microbiol 63:541–556. doi:10.1146/annurev.micro.62.081307.162918. 19575558

[29] Timm CM, Pelletier DA, Jawdy SS, Gunter LE, Henning JA, Engle N, Aufrecht J, Gee E, Nookaew I, Yang Z, Lu T-Y, Tschaplinksi TJ, Doktycz MJ, Tuskan GA, Weston DJ 2016 Two poplar-associated bacterial isolates induce additive favorable responses in a constructed plant-microbiome system. Front Plant Sci 7:497. 27200001 10.3389/fpls.2016.00497 PMC4845692

[30] Weston DJ, Pelletier DA, Morrell-Falvey JL, Tschaplinski TJ, Jawdy SS, Lu TY, Allen SM, Melton SJ, Martin MZ, Schadt CW, Karve AA, Chen JG, Yang X, Doktycz MJ, Tuskan GA 2012 Pseudomonas fluorescens induces strain-dependent and strain-independent host plant responses in defense networks, primary metabolism, photosynthesis, and fitness. Mol Plant Microbe Interact 25:765–778. doi:10.1094/MPMI-09-11-0253. 22375709

[31] Ikeda S, Kaneko T, Okubo T, Rallos LE, Eda S, Mitsui H, Sato S, Nakamura Y, Tabata S, Minamisawa K 2009 Development of a bacterial cell enrichment method and its application to the community analysis in soybean stems. Microb Ecol 58:703–714. doi:10.1007/s00248-009-9566-0. 19662454

[32] Ikeda S, Okubo T, Anda M, Nakashita H, Yasuda M, Sato S, Kaneko T, Tabata S, Eda S, Momiyama A, Terasawa K, Mitsui H, Minamisawa K 2010 Community- and genome-based views of plant-associated bacteria: plant-bacterial interactions in soybean and rice. Plant Cell Physiol 51:1398–1410. doi:10.1093/pcp/pcq119. 20685969

[33] Lundberg DS, Yourstone S, Mieczkowski P, Jones CD, Dangl JL 2013 Practical innovations for high-throughput amplicon sequencing. Nat Methods 10:999–1002. doi:10.1038/nmeth.2634. 23995388

[34] Caporaso JG, Kuczynski J, Stombaugh J, Bittinger K, Bushman FD, Costello EK, Fierer N, Pena AG, Goodrich JK, Gordon JI, Huttley GA, Kelley ST, Knights D, Koenig JE, Ley RE, Lozupone CA, McDonald D, Muegge BD, Pirrung M, Reeder J, Sevinsky JR, Turnbaugh PJ, Walters WA, Widmann J, Yatsunenko T, Zaneveld J, Knight R 2010 QIIME allows analysis of high-throughput community sequencing data. Nat Methods 7:335–336. doi:10.1038/nmeth.f.303. 20383131 PMC3156573

[35] Edgar RC 2013 UPARSE: highly accurate OTU sequences from microbial amplicon reads. Nat Methods 10:996–998. doi:10.1038/nmeth.2604. 23955772

[36] Andrei AS, Robeson MS Jr, Baricz A, Coman C, Muntean V, Ionescu A, Etiope G, Alexe M, Sicora CI, Podar M, Banciu HL 2015 Contrasting taxonomic stratification of microbial communities in two hypersaline meromictic lakes. ISME J 9:2642–2656. doi:10.1038/ismej.2015.60. 25932617 PMC4817630

[37] Campbell AG, Campbell JH, Schwientek P, Woyke T, Sczyrba A, Allman S, Beall CJ, Griffen A, Leys E, Podar M 2013 Multiple single-cell genomes provide insight into functions of uncultured Deltaproteobacteria in the human oral cavity. PLoS One 8:e59361. doi:10.1371/journal.pone.0059361. 23555659 PMC3608642

[38] Wang Q, Garrity GM, Tiedje JM, Cole JR 2007 Naive Bayesian classifier for rapid assignment of rRNA sequences into the new bacterial taxonomy. Appl Environ Microbiol 73:5261–5267. doi:10.1128/AEM.00062-07. 17586664 PMC1950982

[39] Pruesse E, Peplies J, Glockner FO 2012 SINA: accurate high-throughput multiple sequence alignment of ribosomal RNA genes. Bioinformatics 28:1823–1829. doi:10.1093/bioinformatics/bts252. 22556368 PMC3389763

[40] Brown CT, Howe A, Zhang Q, Pyrkosz AB, Brom TH 2012 A reference-free algorithm for computational normalization of shotgun sequencing data. arXiv:1203.4802v2.

[41] Utturkar SM, Klingeman DM, Land ML, Schadt CW, Doktycz MJ, Pelletier DA, Brown SD 2014 Evaluation and validation of *de novo* and hybrid assembly techniques to derive high quality genome sequences. Bioinformatics 30:2709–2716. doi:10.1093/bioinformatics/btu391. 24930142 PMC4173024

[42] CLC. 2015 CLC Genomics Workbench manual: trimming using the Trim tool. http://www.clcsupport.com/clcgenomicsworkbench/800/index.php?manual=Trimming_using_Trim_tool.html.

[43] Peng Y, Leung HC, Yiu SM, Chin FY 2012 IDBA-UD: a *de novo* assembler for single-cell and metagenomic sequencing data with highly uneven depth. Bioinformatics 28:1420–1428. doi:10.1093/bioinformatics/bts174. 22495754

[44] Bankevich A, Nurk S, Antipov D, Gurevich AA, Dvorkin M, Kulikov AS, Lesin VM, Nikolenko SI, Pham S, Prjibelski AD, Pyshkin AV, Sirotkin AV, Vyahhi N, Tesler G, Alekseyev MA, Pevzner PA 2012 SPAdes: a new genome assembly algorithm and its applications to single-cell sequencing. J Comput Biol 19:455–477. doi:10.1089/cmb.2012.0021. 22506599 PMC3342519

[45] Chitsaz H, Yee-Greenbaum JL, Tesler G, Lombardo MJ, Dupont CL, Badger JH, Novotny M, Rusch DB, Fraser LJ, Gormley NA, Schulz-Trieglaff O, Smith GP, Evers DJ, Pevzner PA, Lasken RS 2011 Efficient *de novo* assembly of single-cell bacterial genomes from short-read data sets. Nat Biotechnol 29:915–921. doi:10.1038/nbt.1966. 21926975 PMC3558281

[46] Joint Genome Institute. 2015 Single cell data decontamination. https://docs.google.com/viewer?a=v&pid=sites&srcid=bGJsLmdvdnxpbWctZm9ybXxneDoxMDUwZTdmYTJiOGQ4ZTAy.

[47] Hyatt D, Chen GL, Locascio PF, Land ML, Larimer FW, Hauser LJ 2010 Prodigal: prokaryotic gene recognition and translation initiation site identification. BMC Bioinformatics 11:119. doi:10.1186/1471-2105-11-119. 20211023 PMC2848648

[48] Markowitz VM, Chen IM, Palaniappan K, Chu K, Szeto E, Grechkin Y, Ratner A, Jacob B, Huang J, Williams P, Huntemann M, Anderson I, Mavromatis K, Ivanova NN, Kyrpides NC 2012 IMG: the Integrated Microbial Genomes database and comparative analysis system. Nucleic Acids Res 40:D115–D122. doi:10.1093/nar/gkr1044. 22194640 PMC3245086

[49] Parks DH, Imelfort M, Skennerton CT, Hugenholtz P, Tyson GW 2015 CheckM: assessing the quality of microbial genomes recovered from isolates, single cells, and metagenomes. Genome Res 25:1043–1055. doi:10.1101/gr.186072.114. 25977477 PMC4484387

[50] Land ML, Hyatt D, Jun SR, Kora GH, Hauser LJ, Lukjancenko O, Ussery DW 2014 Quality scores for 32,000 genomes. Stand Genomic Sci 9:20. doi:10.1186/1944-3277-9-20. 25780509 PMC4334873

[51] Darling AE, Jospin G, Lowe E, Matsen FAt Bik HM, Eisen JA 2014 PhyloSift: phylogenetic analysis of genomes and metagenomes. PeerJ 2:e243. doi:10.7717/peerj.243. 24482762 PMC3897386

[52] Chen IM, Markowitz VM, Chu K, Anderson I, Mavromatis K, Kyrpides NC, Ivanova NN 2013 Improving microbial genome annotations in an integrated database context. PLoS One 8:e54859. doi:10.1371/journal.pone.0054859. 23424620 PMC3570495

[53] Joint Genome Institute. 2015 IMG Pfam Clans. https://img.jgi.doe.gov/cgi-bin/er/main.cgi?section=FindFunctions&page=pfamListClans.

[54] Joint Genome Institute. 2015 IMG COG categories. https://img.jgi.doe.gov/cgi-bin/er/main.cgi?section=FindFunctions&page=cogid2cat.

[55] Salter SJ, Cox MJ, Turek EM, Calus ST, Cookson WO, Moffatt MF, Turner P, Parkhill J, Loman NJ, Walker AW 2014 Reagent and laboratory contamination can critically impact sequence-based microbiome analyses. BMC Biol 12:87. doi:10.1186/s12915-014-0087-z. 25387460 PMC4228153

[56] Tennessen K, Andersen E, Clingenpeel S, Rinke C, Lundberg DS, Han J, Dangl JL, Ivanova N, Woyke T, Kyrpides N, Pati A 2016 ProDeGe: a computational protocol for fully automated decontamination of genomes. ISME J 10:269–272. doi:10.1038/ismej.2015.100. 26057843 PMC4681846

[57] Wu D, Hugenholtz P, Mavromatis K, Pukall R, Dalin E, Ivanova NN, Kunin V, Goodwin L, Wu M, Tindall BJ, Hooper SD, Pati A, Lykidis A, Spring S, Anderson IJ, D'Haeseleer P, Zemla A, Singer M, Lapidus A, Nolan M, Copeland A, Han C, Chen F, Cheng JF, Lucas S, Kerfeld C, Lang E, Gronow S, Chain P, Bruce D, Rubin EM, Kyrpides NC, Klenk HP, Eisen JA 2009 A phylogeny-driven genomic encyclopedia of Bacteria and Archaea. Nature 462:1056–1060. doi:10.1038/nature08656. 20033048 PMC3073058

[58] Zaneveld JR, Lozupone C, Gordon JI, Knight R 2010 Ribosomal RNA diversity predicts genome diversity in gut bacteria and their relatives. Nucleic Acids Res 38:3869–3879. doi:10.1093/nar/gkq066. 20197316 PMC2896507

[59] Szöllosi GJ, Boussau B, Abby SS, Tannier E, Daubin V 2012 Phylogenetic modeling of lateral gene transfer reconstructs the pattern and relative timing of speciations. Proc Natl Acad Sci U S A 109:17513–17518. doi:10.1073/pnas.1202997109. 23043116 PMC3491530

[60] Cole JR, Wang Q, Fish JA, Chai B, McGarrell DM, Sun Y, Brown CT, Porras-Alfaro A, Kuske CR, Tiedje JM 2014 Ribosomal database project: data and tools for high-throughput rRNA analysis. Nucleic Acids Res 42:D633–D642. doi:10.1093/nar/gkt1244. 24288368 PMC3965039

[61] Kantor RS, Wrighton KC, Handley KM, Sharon I, Hug LA, Castelle CJ, Thomas BC, Banfield JF 2013 Small genomes and sparse metabolisms of sediment-associated bacteria from four candidate phyla. mBio 4:. doi:10.1128/mBio.00708-13. PMC3812714 24149512

[62] Wrighton KC, Thomas BC, Sharon I, Miller CS, Castelle CJ, VerBerkmoes NC, Wilkins MJ, Hettich RL, Lipton MS, Williams KH, Long PE, Banfield JF 2012 Fermentation, hydrogen, and sulfur metabolism in multiple uncultivated bacterial phyla. Science 337:1661–1665. doi:10.1126/science.1224041. 23019650

[63] Brewer T, Handley K, Carini P, Gibert J, Fierer N 18 5 2016 Genome reduction in an abundant and ubiquitous soil bacterial lineage. bioRxiv doi:10.1101/053942. 27798560

[64] Hu ZY, Wang YZ, Im WT, Wang SY, Zhao GP, Zheng HJ, Quan ZX 2014 The first complete genome sequence of the class Fimbriimonadia in the phylum Armatimonadetes. PLoS One 9:e100794. doi:10.1371/journal.pone.0100794. 24967843 PMC4072686

[65] Lee KC, Morgan XC, Dunfield PF, Tamas I, McDonald IR, Stott MB 2014 Genomic analysis of Chthonomonas calidirosea, the first sequenced isolate of the phylum Armatimonadetes. ISME J 8:1522–1533. doi:10.1038/ismej.2013.251. 24477196 PMC4069393

[66] Lin S, Cronan JE 2011 Closing in on complete pathways of biotin biosynthesis. Mol Biosyst 7:1811–1821. doi:10.1039/c1mb05022b. 21437340

[67] Rodionov DA, Mironov AA, Gelfand MS 2002 Conservation of the biotin regulon and the BirA regulatory signal in Eubacteria and Archaea. Genome Res 12:1507–1516. doi:10.1101/gr.314502. 12368242 PMC187538

[68] Guo M, Han X, Jin T, Zhou L, Yang J, Li Z, Chen J, Geng B, Zou Y, Wan D, Li D, Dai W, Wang H, Chen Y, Ni P, Fang C, Yang R 2012 Genome sequences of three species in the family Planctomycetaceae. J Bacteriol 194:3740–3741. doi:10.1128/JB.00639-12. 22740668 PMC3393480

[69] Lv J, Jiang Y, Yu Q, Lu S 2011 Structural and functional role of nickel ions in urease by molecular dynamics simulation. J Biol Inorg Chem 16:125–135. doi:10.1007/s00775-010-0711-5. 20890717

[70] Zhang Y, Rodionov DA, Gelfand MS, Gladyshev VN 2009 Comparative genomic analyses of nickel, cobalt and vitamin B12 utilization. BMC Genomics 10:78. doi:10.1186/1471-2164-10-78. 19208259 PMC2667541

[71] Cotter PD, Hill C 2003 Surviving the acid test: responses of Gram-positive bacteria to low pH. Microbiol Mol Biol Rev 67:429–453. doi:10.1128/MMBR.67.3.429-453.2003. 12966143 PMC193868

[72] Richard HT, Foster JW 2003 Acid resistance in Escherichia coli. Adv Appl Microbiol 52:167–186. doi:10.1016/S0065-2164(03)01007-4. 12964244

[73] Richard H, Foster JW 2004 Escherichia coli glutamate- and arginine-dependent acid resistance systems increase internal pH and reverse transmembrane potential. J Bacteriol 186:6032–6041. doi:10.1128/JB.186.18.6032-6041.2004. 15342572 PMC515135

[74] Stingl K, Altendorf K, Bakker EP 2002 Acid survival of Helicobacter pylori: how does urease activity trigger cytoplasmic pH homeostasis? Trends Microbiol 10:70–74. doi:10.1016/S0966-842X(01)02287-9. 11827807

[75] Wilson CM, Loach D, Lawley B, Bell T, Sims IM, O'Toole PW, Zomer A, Tannock GW 2014 Lactobacillus reuteri 100-23 modulates urea hydrolysis in the murine stomach. Appl Environ Microbiol 80:6104–6113. doi:10.1128/AEM.01876-14. 25063664 PMC4178674

[76] Kulichevskaya IS, Baulina OI, Bodelier PL, Rijpstra WI, Damste JS, Dedysh SN 2009 Zavarzinella formosa gen. nov., sp. nov., a novel stalked, Gemmata-like planctomycete from a Siberian peat bog. Int J Syst Evol Microbiol 59:357–364. doi:10.1099/ijs.0.002378-0. 19196778

[77] Foster RC, Bowen GD 1982 Plant surfaces and bacterial growth: the rhizosphere and rhizoplane, p 159–185. *In* Mount M, Lacy GH (ed), Phytopathogenic prokaryotes. Elsevier, New York, NY.

[78] Kant R, van Passel MW, Palva A, Lucas S, Lapidus A, Glavina del Rio T, Dalin E, Tice H, Bruce D, Goodwin L, Pitluck S, Larimer FW, Land ML, Hauser L, Sangwan P, de Vos WM, Janssen PH, Smidt H 2011 Genome sequence of Chthoniobacter flavus Ellin428, an aerobic heterotrophic soil bacterium. J Bacteriol 193:2902–2903. doi:10.1128/JB.00295-11. 21460085 PMC3133135

[79] Sangwan P, Chen X, Hugenholtz P, Janssen PH 2004 Chthoniobacter flavus gen. nov., sp. nov., the first pure-culture representative of subdivision two, Spartobacteria classis nov., of the phylum Verrucomicrobia. Appl Environ Microbiol 70:5875–5881. doi:10.1128/AEM.70.10.5875-5881.2004. 15466527 PMC522106

[80] Omsland A, Cockrell DC, Howe D, Fischer ER, Virtaneva K, Sturdevant DE, Porcella SF, Heinzen RA 2009 Host cell-free growth of the Q fever bacterium Coxiella burnetii. Proc Natl Acad Sci U S A 106:4430–4434. doi:10.1073/pnas.0812074106. 19246385 PMC2657411

[81] Wang X, Sharp CE, Jones GM, Grasby SE, Brady AL, Dunfield PF 2015 Stable-isotope probing identifies uncultured Planctomycetes as primary degraders of a complex heteropolysaccharide in soil. Appl Environ Microbiol 81:4607–4615. doi:10.1128/AEM.00055-15. 25934620 PMC4551180

[82] Dunfield PF, Tamas I, Lee KC, Morgan XC, McDonald IR, Stott MB 2012 Electing a candidate: a speculative history of the bacterial phylum OP10. Environ Microbiol 14:3069–3080. doi:10.1111/j.1462-2920.2012.02742.x. 22497633

