# Supplementary material for: Enrichment of Root Endophytic Bacteria from Populus deltoides and Single-Cell-Genomics Analysis
Source: Appl Environ Microbiol. 2016 Aug 30;82(18):5698–708. doi: 10.1128/AEM.01285-16 (PMC5007785; doi:10.1128/AEM.01285-16)
Supplement: Supplemental material [file supp_82_18_5698__index.html]

Supplemental material 

# Enrichment of Root Endophytic Bacteria from Populus deltoides and Single-Cell-Genomics Analysis

## Supplemental material

- Supplemental file 1 -

  Presence of conserved marker genes in SAGs and selected reference strains used for phylogenetic analyses (Table S1) and summary of genome completeness quality scores for each SAG (Table S2).

  XLSX, 12K
